# Decrease in acetyl-CoA pathway utilizing butyrate-producing bacteria is a key pathogenic feature of alcohol-induced functional gut microbial dysbiosis and development of liver disease in mice

**DOI:** 10.1080/19490976.2021.1946367

**Published:** 2021-08-07

**Authors:** Richa Singhal, Hridgandh Donde, Smita Ghare, Kendall Stocke, Jingwein Zhang, Manicka Vadhanam, Sreelatha Reddy, Leila Gobejishvili, Paula Chilton, Swati Joshi-Barve, Wenke Feng, Craig McClain, Kristi Hoffman, Joseph Petrosino, Marius Vital, Shirish Barve

**Affiliations:** a University of Louisville Department of Medicine; b University of Louisville Alcohol Research Center; c University of Louisville Department of Environmental and Occupational Health Science; d Baylor College of Medicine Department of Molecular Virology and Microbiology; e Baylor College of Medicine Center for Metagenomics and Microbiome Research; f Hannover Medical School, Hanover, Germany; g Helmholtz Center for Infection Research; h Helmholtz Association of German Research Centers; i University of Louisville Department of Pharmacology and Toxicology

**Keywords:** Butyrate, alcohol, butyrate pathways, Ruminococcaceae, Lachnospiraceae, acetyl-CoA, tributyrin, alcohol-associated liver disease

## Abstract

Emerging research evidence has established the critical role of the gut-liver axis in the development of alcohol-associated liver disease (ALD). The present study employed 16S rRNA gene and whole genome shotgun (WGS) metagenomic analysis in combination with a revised microbial dataset to comprehensively detail the butyrate-producing microbial communities and the associated butyrate metabolic pathways affected by chronic ethanol feeding. Specifically, the data demonstrated that a decrease in several butyrate-producing bacterial genera belonging to distinct families within the Firmicutes phyla was a significant component of ethanol-induced dysbiosis. WGS analysis of total bacterial genomes encompassing butyrate synthesizing pathways provided the functional characteristics of the microbiome associated with butyrate synthesis. The data revealed that in control mice microbiome, the acetyl-coenzyme A (CoA) butyrate synthesizing pathway was the most prevalent and was significantly and maximally decreased by chronic ethanol feeding. Further WGS analysis i) validated the ethanol-induced decrease in the acetyl-CoA pathway by identifying the decrease in two critical genes but – (butyryl-CoA: acetate CoA transferase) and buk – (butyrate kinase) that encode the terminal condensing enzymes required for converting butyryl-CoA to butyrate and ii) detection of specific taxa of butyrate-producing bacteria containing but and buk genes. Notably, the administration of tributyrin (Tb) – a butyrate prodrug - significantly prevented ethanol-induced decrease in butyrate-producing bacteria, hepatic steatosis, inflammation, and injury. Taken together, our findings strongly suggest that the loss of butyrate-producing bacteria using the acetyl-CoA pathway is a significant pathogenic feature of ethanol-induced microbial dysbiosis and ALD and can be targeted for therapy.

## Introduction

Alterations in the gut microbiome (dysbiosis) are increasingly recognized as a major pathogenic factor in the development and progression of alcohol-associated liver disease associated with alcohol-related human disorders.^1–4^ Clinical and pre-clinical studies done by our research group and others using bacterial 16S rRNA gene sequencing have shown that chronic alcohol consumption leads to enteric dysbiosis, involving qualitative and quantitative changes in the intestinal microbiome that consequently leads to the development of liver disease.^1,5,6^ Although these studies using 16S rRNA gene sequencing provided information regarding bacterial phylogeny and taxonomy, they did not reveal the functional potential of the microbiota and lacked the resolution of species or strain level.

An important function of the gut microbiome is the fermentation of indigestible dietary fibers leading to the production of short-chain fatty acids (SCFAs).^7^ Among the SCFAs produced by the gut microbiome, butyrate has been demonstrated to confer multiple health benefits to the host.^7^ Hence, depending on the type and extent of microbial dysbiosis, normal production of butyrate and consequent health benefits could be negatively impacted. With regard to the effects of alcohol on the gut microbiome and the development of liver disease, studies have demonstrated that i) chronic exposure to ethanol leads to a decrease in intestinal butyrate levels^8,9^ and ii) administration of tributyrin (Tb) – a butyrate prodrug significantly attenuates ethanol mediated intestinal barrier dysfunction and hepatic steatosis and injury.^10–12^ These findings clearly indicate that alcohol-induced gut dysbiosis likely affects the butyrate-producing microbial communities. Therefore, the major goal of the present study was to elucidate the effects of chronic ethanol consumption on butyrate synthesizing microbial communities and the associated butyrate synthesizing pathways.

Specifically, we profiled the fecal microbiome using 16S rRNA sequencing and Whole Genome Shotgun (WGS) metagenomics sequencing along with inferred metagenomics using PICRUSt.^13,14^ The data obtained reveal the impact of chronic ethanol on the diversity of butyrate-producing bacterial communities along with changes in the butyrate synthesizing bacterial genes and their involvement in butyrate synthesis pathways. Moreover, the WGS analysis led to the identification of relevant butyrate-producing bacteria affected by ethanol, at the species level. Importantly, the present work also investigated the pathogenic relevance of ethanol-induced loss of butyrate-producing bacteria by examining the effects of oral administration of Tb on the microbial dysbiosis and development of liver disease – hepatic steatosis, inflammation, and injury.

## Results

### Chronic ethanol feeding leads to a decrease in gut microbial diversity

**Figure 1.**
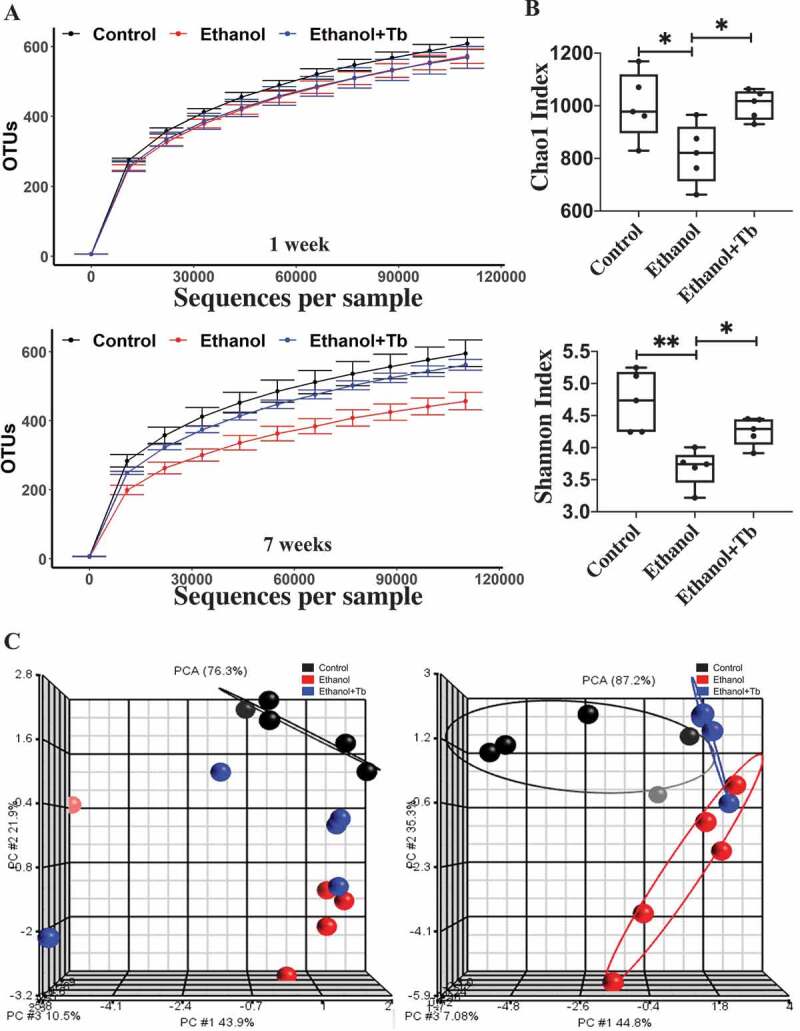
Reduced microbial diversity and distinct microbial composition observed in chronic ethanol-fed mice through 16S analysis. A) Alpha rarefaction curves for one-week and seven-week treatments, B) Alpha diversity indices are shown – Chao 1 Index measuring species richness (left) and Shannon Index measuring species abundance and evenness (right). Data represented as mean ± SEM for n = 5 in each treatment group with one-way ANOVA Tukey's corrected *p*-value, *p* < .05 = * and *p* < .01 = ** and C) Beta diversity (depicting compositional differences) PCA plots generated using Weighted Unifrac distances for one-week and seven-week treatments

The effect of chronic ethanol feeding and Tb oral administration on the gut microbial diversity was assessed. In the initial phase of the chronic ethanol feeding (1 week), alpha rarefaction curves representing within sample variation were not significantly different between treatment groups (Figure 1a). In comparison, after 7 weeks, ethanol feeding resulted in a curve that saturates at a lower point than control, indicating a decrease in microbial diversity over time (Figure 1a). Examination of the effects of Tb administration on ethanol-induced changes in the gut microbiome showed that alpha rarefaction curves remained unchanged between Control and Ethanol+Tb animals. These data indicate that Tb administration prevented the ethanol-induced loss of microbial diversity. Further, alpha diversity indices, Chao1 (measuring species richness), and Shannon Index (measuring species abundance and evenness) were also examined at 7 weeks. The data showed that in comparison with Control, there was a significant decrease in both diversity indices in the ethanol-fed group (Figure 1b). This ethanol-induced decrease in diversity was significantly prevented by Tb administration (Figure 1b). Overall, the predictive alpha diversity measures indicate that chronic ethanol feeding, over time, significantly decreases microbial diversity, which can be counteracted by Tb administration.

Further, beta diversity was assessed by examining the dissimilarity of the microbial taxa between the treatment groups, using the phylogeny based Weighted Unifrac distance metric principal component analysis (PCA) plots. At 1 week, PCA plots demonstrated that Tb administration did not have a significant impact on the ethanol-induced changes in the gut microbial composition (Figure 1c). However, after 7 weeks of Tb administration, Ethanol, and Ethanol + Tb groups clustered into distinct treatment groups. These data indicate that ethanol can initiate perturbations in the microbial composition in as early as 1 week of chronic ethanol feeding, which continues to increase over a period of 7 weeks. Importantly, ethanol-induced changes in microbial communities are mitigated by Tb administration, preventing microbial dysbiosis that increases over time (7 weeks).

### Ethanol-induced dysbiosis is characterized by a decrease in butyrate-producing bacteria

**Figure 2.**
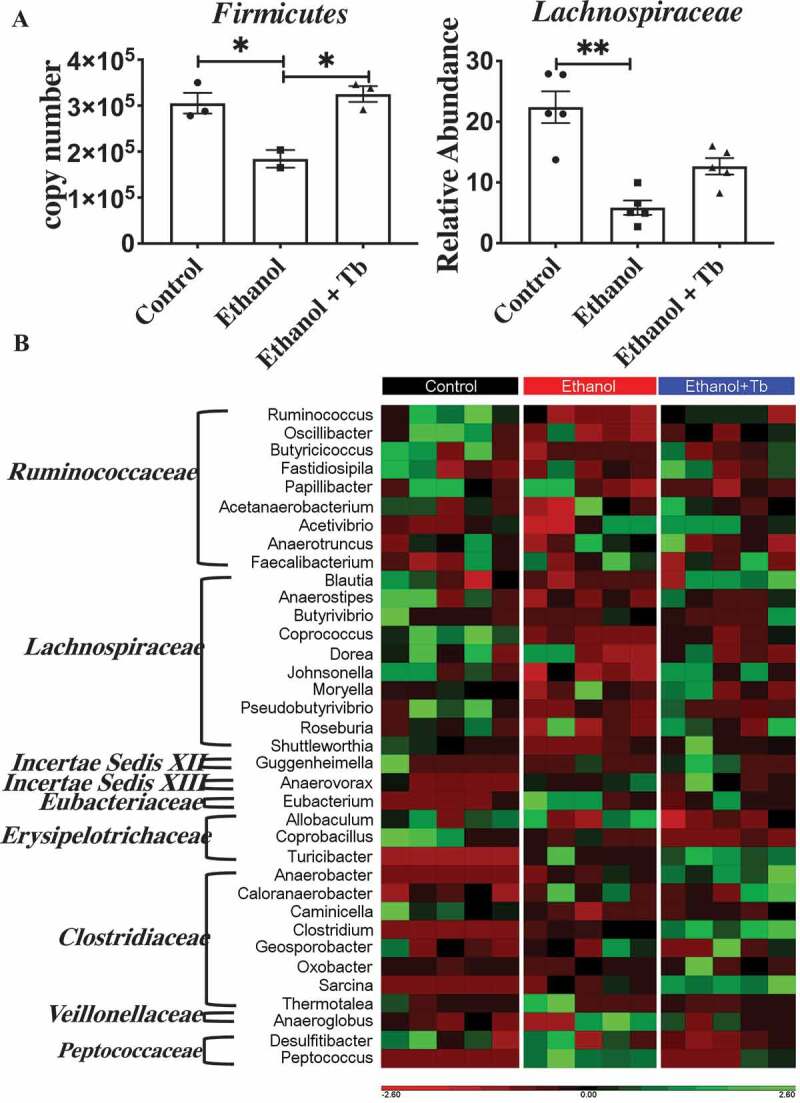
Reduction in relative abundance of butyrate-producing taxa observed in chronic ethanol-fed mice. A) Significant decrease in relative abundance of *Firmicutes* (left) and *Lachnospiraceae* family (right). Data represented as mean ± SEM for n ≤ 5 mice in each treatment group along with Kruskal Wallis Dunn’s corrected *p*-value, *p* < .05 = * and *p* < .01 = ** and B). Heat map illustrating the effect of different treatment groups (Control, Ethanol, and Ethanol +Tb) on 36 different butyrate-producing genera belonging to *Firmicutes* families identified by 16S sequencing. Red color signifies that genus is present in low abundance or absent and green color signifies that genus is highly abundant

In accordance with, our earlier work, the present data demonstrated that chronic ethanol feeding induced gut microbial dysbiosis involves a significant decrease in the phylum *Firmicutes*^5^ (Figure 2a). Importantly, the phylum *Firmicutes* harbors the majority of butyrate-producing microbial families and genera and it has been documented that in the gut, alcohol decreases the levels of short-chain fatty acids (SCFAs), particularly butyrate.^8,9,15^ Since the decline in butyrate likely occurs due to ethanol-induced gut microbial dysbiosis represented by a decline in the phylum *Firmicutes*, we examined the status of the butyrate-producing bacterial communities in this phylum. Among all the butyrate-producing families within *Firmicutes*, we identified that ethanol feeding caused a substantial decrease in the largest butyrate-producing family *Lachnospiraceace* (Figure 2a). Further, we determined the impact of chronic ethanol feeding on 36 distinct butyrate-producing genera belonging to these eight families by employing the catalog of butyrate-producing bacteria provided by Vital et al. 2017^15^ and additional literature review detailed in Supplemental Table SI.

Compared to control, ethanol feeding led to a significant decline in the relative abundance of 13 potential butyrate-producing genera across four families. Specifically, I) *Lachnospiraceace* family – involving genera *Anaerostipes, Coprococcus, Roseburia, Dorea, Johnsonella, Shuttleworthia, and Pseudobutyrivibrio*, II) *Ruminococccaceae* family – involving genera *Ruminococcus, Oscillibacter*, and *Butyricicoccus* III) *Clostridiaceae* family – involving genus *Caminicella*, IV) *Erysipelotrichaceae* family – involving genus *Coprobacillus* (Table 1; Ethanol vs. Control). Interestingly, Tb besides functioning as a butyrate prodrug, also prevented the ethanol-induced decrease in 12 out of 13 of the butyrate-producing bacterial genera in comparison to the control group (Table 1; Ethanol + Tb vs. Control). Further, comparison of Ethanol vs. Ethanol + Tb (Table 1) also showed that the negative effect of ethanol on butyrate producing genera was mitigated by Tb. Collectively, these results demonstrate that a significant component of ethanol-induced dysbiosis involves a decrease in several butyrate-producing bacterial genera, which can be prevented by Tb supplementation.

**Table 1.** Ethanol-induced reduction in butyrate-producing genera

Family	Genera	Ethanol vs. Control		Ethanol+Tb vs. Control		Ethanol vs.Ethanol+Tb	
		Trend	Significance	Trend	Significance	Trend	Significance
*Ruminococcaceae*	*Ruminococcus*	↓	**	↓	NS	↓	NS
*Ruminococcaceae*	*Oscillibacter*	↓	*	↓	NS	↓	NS
*Ruminococcaceae*	*Butyricicoccus*	↓	*	↓	NS	↓	NS
*Ruminococcaceae*	*Fastidiosipila*	↓	NS	↑	NS	↓	NS
*Ruminococcaceae*	*Papillibacter*	↓	Trending	↓	NS	↑	NS
*Ruminococcaceae*	*Acetanaerobacterium*	↓	Trending	↑	NS	↓	NS
*Ruminococcaceae*	*Acetivibrio*	↑	Trending	↑	NS	↓	NS
*Ruminococcaceae*	*Anaerotruncus*	↓	NS	↓	NS	↑	NS
*Ruminococcaceae*	*Faecalibacterium*	↑	Trending	↑	NS	↑	NS
*Lachnospiraceae*	*Blautia*	↓	Trending	↑	NS	↓	NS
*Lachnospiraceae*	*Anaerostipes*	↓	*	↓	NS	↓	NS
*Lachnospiraceae*	*Butyrivibrio*	↓	Trending	↓	NS	↓	NS
*Lachnospiraceae*	*Coprococcus*	↓	**	↓	Trending	↓	NS
*Lachnospiraceae*	*Dorea*	↓	*	↓	NS	↑	NS
*Lachnospiraceae*	*Johnsonella*	↓	*	↑	NS	↓	NS
*Lachnospiraceae*	*Moryella*	↑	Trending	↑	NS	↑	NS
*Lachnospiraceae*	*Pseudobutyrivibrio*	↓	**	↓	NS	↓	NS
*Lachnospiraceae*	*Roseburia*	↓	*	↑	NS	↓	NS
*Lachnospiraceae*	*Shuttleworthia*	↓	**	↑	NS	↓	NS
*Incertae Sedis XII*	*Guggenheimella*	↓	Trending	↑	NS	↓	NS
*Incertae Sedis XIII*	*Anaerovorax*	↑	**	↑	NS	↓	NS
*Eubacteriaceae*	*Eubacterium*	↑	**	↑	NS	↑	NS
*Erysipelotrichaceae*	*Allobaculum*	↑	NS	↓	NS	↑	NS
*Erysipelotrichaceae*	*Coprobacillus*	↓	*	↓	**	↑	NS
*Erysipelotrichaceae*	*Turicibacter*	↑	*	↑	**	↓	NS
*Clostridiaceae*	*Anaerobacter*	↑	*	↑	**	↓	NS
*Clostridiaceae*	*Caloranaerobacter*	↑	Trending	↑	NS	↓	NS
*Clostridiaceae*	*Caminicella*	↓	**	↓	Trending	↓	NS
*Clostridiaceae*	*Clostridium*	↑	*	↑	**	↓	NS
*Clostridiaceae*	*Geosporobacter*	↑	Trending	↑	NS	↓	NS
*Clostridiaceae*	*Oxobacter*	↑	NS	↑	NS	↓	NS
*Clostridiaceae*	*Sarcina*	↑	*	↑	**	↓	NS
*Clostridiaceae*	*Thermotalea*	↑	Trending	↓	NS	↓	NS
*Veillonellaceae*	*Anaeroglobus*	↑	Trending	↑	NS	↓	NS
*Peptococcaceae*	*Desulfitibacter*	↓	NS	↓	NS	↓	NS
*Peptococcaceae*	*Peptococcus*	↑	**	↑	NS	↓	NS

Kruskal Wallis test comparing relative abundance of butyrate-producing genera between Ethanol vs. Control, Ethanol + Tb vs. Control and Ethanol vs. Ethanol+Tb. NS – no significance, q-value < 0.05 = *, q- value < 0.01 = ** and Trending = (q-value between 0.051–0.08)

### Predicted functional profiles of butyrate synthesizing genes affected by ethanol and tributyrin

**Figure 3.**
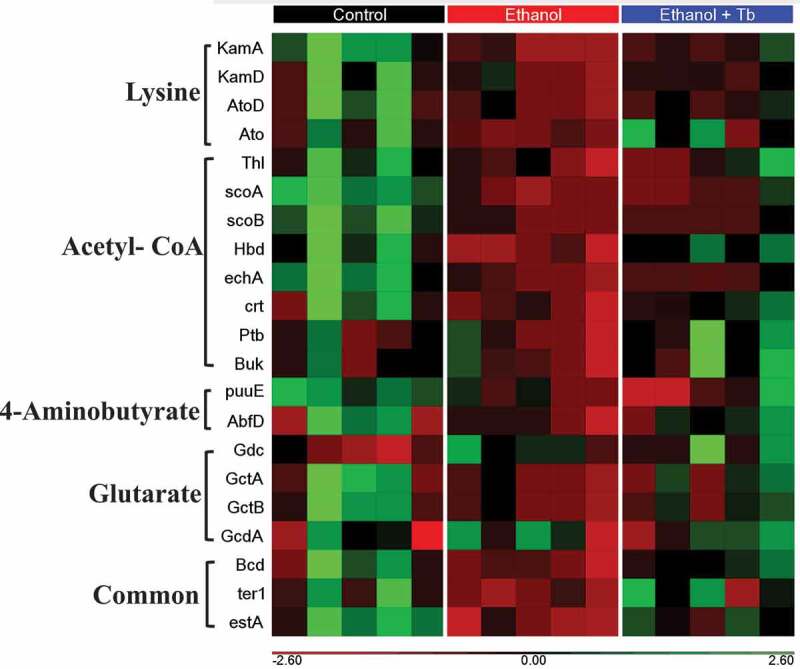
Reduction in the abundance of butyrate synthesizing genes observed in chronic ethanol-fed mice. Heat map generated for 21 distinct butyrate synthesis genes identified by PICRUSt for Control, Ethanol, and Ethanol + Tb treatments. A heat map shows genes from lysine, acetyl-CoA, 4-aminobutyrate, glutarate, Common (all four pathways), and putative tributyrin esterase that hydrolysis Tb to produce butyrate. Red color signifies that genes are present in low abundance or absent and green color signifies that genes are highly abundant. *KamA* – lysine-2,3-aminomutase, *KamD* – β-lysine-5,6-aminomutase α, *AtoD* -butyryl-CoA:acetoacetate CoA transferase (α subunit), *Ato* – butyrate-acetoacetate CoA-transferase, *Thl –* thiolase, *scoA* – 3-oxoacid CoA-transferase subunit A, *scoB* −3-oxoacid CoA-transferase subunit B, *Hbd*- β-hydroxybutyryl-CoA dehydrogenase, *echA* & *crt –* crotonase, *Ptb* – phosphate butyryltransferase, *Buk*- butyrate kinase, *puuE* 4-aminobutyrate transaminase, *Gdc* – glutamate decarboxylase, *GctA* -glutaconate CoA transferase (α), *GctB* – glutaconate CoA transferase (β), *GcdA* – glutaconyl-CoA decarboxylase (α, β subunits), *Bcd –* butyryl-CoA dehydrogenase (including electron transfer protein α, β subunits, *ter1*-trans-2-enoyl-CoA reductase (NAD+), *estA*- putative tributyrin esterase

Following taxonomic analyses, we examined the effects of ethanol and Tb on the functional characteristics of the microbiome associated with butyrate synthesis using inferred metagenomics. Putative determination of butyrate synthesizing genes was achieved from PICRUSt output based on in-house updated butyrate genes inventory (Supplemental Table SII). This inventory was generated by using functional aspects provided by KEGG Orthology pertaining to various facets of butyrate synthesis and as previously described by Vital *et al*. 2013.^15^ This predictive analysis showed that the gut microbiome of ethanol-treated animals had a significant reduction in the abundance of butyrate synthesizing genes associated with the four-butyrate synthesizing pathways, namely, acetyl-CoA, lysine, 4-aminobutyrate, and glutarate pathway (Figure 3a and Table 2). Moreover, the putative tributyrin esterase (*estA*) gene that hydrolyzes Tb to generate butyrate was also predicted to be significantly reduced due to chronic ethanol feeding, indicating that ethanol potentially decreases the capacity of the gut microbiome to derive butyrate from dietary Tb (Figure 3a and Table 2). A reduction in cecal butyrate levels validated the predicted decrease in butyrate synthesizing capacity of the gut microbiome in ethanol-fed mice (Supplemental Figure S1). Further, a Taxa PICRUST analysis was performed to qualitatively assign the decrease in butyrate synthesizing genes to bacterial families. This analysis showed that *Lachnospiraceace* and *Ruminococcaceae* were the predominant families that potentially contributed toward the ethanol-induced decrease in butyrate synthesizing genes in all four pathways (Supplemental Figure S2). Interestingly, Tb supplementation significantly prevented this ethanol-induced decrease in butyrate synthesizing genes (Table 2; Supplemental Figure S2).

**Table 2.** Ethanol-induced reduction in butyrate synthesizing genes

Abbreviation	Name	Butyrate Pathway	Ethanol vs. Control		Ethanol + Tb vs. Control		Ethanol vs. Ethanol+Tb	
			Trend	Significance	Trend	Significance	Trend	Significance
*KamA*	lysine-2,3-aminomutase	Lysine	↓	**	↓	NS	↓	NS
*KamD*	β-lysine-5,6-aminomutase α	Lysine	↓	**	↓	NS	↓	NS
*AtoD*	butyryl-CoA:acetoacetate CoA transferase (α subunit)	Lysine	↓	**	↓	NS	↓	NS
*Ato*	butyrate-acetoacetate CoA-transferase	Lysine	↓	**	No change	NS	↓	Trending
*Thl*	thiolase	Acetyl-CoA	↓	**	↓	NS	↓	NS
*scoA*	3-oxoacid CoA-transferase subunit A	Acetyl-CoA	↓	**	↓	NS	↓	NS
*scoB*	3-oxoacid CoA-transferase subunit B	Acetyl-CoA	↓	**	↓	NS	↓	NS
*Hbd*	β-hydroxybutyryl-CoA dehydrogenase	Acetyl-CoA	↓	**	↓	NS	↓	NS
*echA*	crotonase	Acetyl-CoA	↓	**	↓	NS	↓	NS
*crt*	crotonase	Acetyl-CoA	↓	**	↓	NS	↓	Trending
*Ptb*	phosphate butyryltransferase	Acetyl-CoA	↓	*	↑	NS	↓	Trending
*Buk*	butyrate kinase	Acetyl-CoA	↓	*	↑	NS	↓	Trending
*puuE*	4-aminobutyrate transaminase	4 Aminobutyrate	↓	**	↓	NS	↑	NS
*AbfD*	4-hydroxybutyryl-CoA dehydratase	4 Aminobutyrate	↓	*	↓	NS	↓	NS
*Gdc*	Glutamate decarboxylase	Glutarate	↑	**	↑	NS	↓	NS
*GctA*	glutaconate CoA transferase (α)	Glutarate	↓	**	↓	NS	↓	NS
*GctB*	glutaconate CoA transferase (β)	Glutarate	↓	**	↓	NS	↓	NS
*GcdA*	glutaconyl-CoA decarboxylase (α, β subunits)	Glutarate	↑	*	↑	NS	↑	NS
*Bcd*	butyryl-CoA dehydrogenase (including ETP α, β subunits)	Common to all	↓	**	↓	NS	↓	Trending
*ter1*	trans-2-enoyl-CoA reductase (NAD+)	Common to all	↓	**	↓	NS	↓	Trending
estA	putative tributyrin esterase	Hydrolysis of tributyrin	↓	**	↓	NS	↓	Trending

Kruskal Wallis test comparing abundance of butyrate synthesizing genes between – Ethanol vs. Control, Ethanol + Tb vs. Control and Ethanol vs. Ethanol+Tb. NS – no significance, q-value < 0.05 = *, q- value < 0.01 = ** and Trending = (q-value between 0.051–0.08).

### Whole genome shotgun (WGS) analysis of butyrate synthesizing pathways affected by ethanol and tributyrin

**Figure 4.**
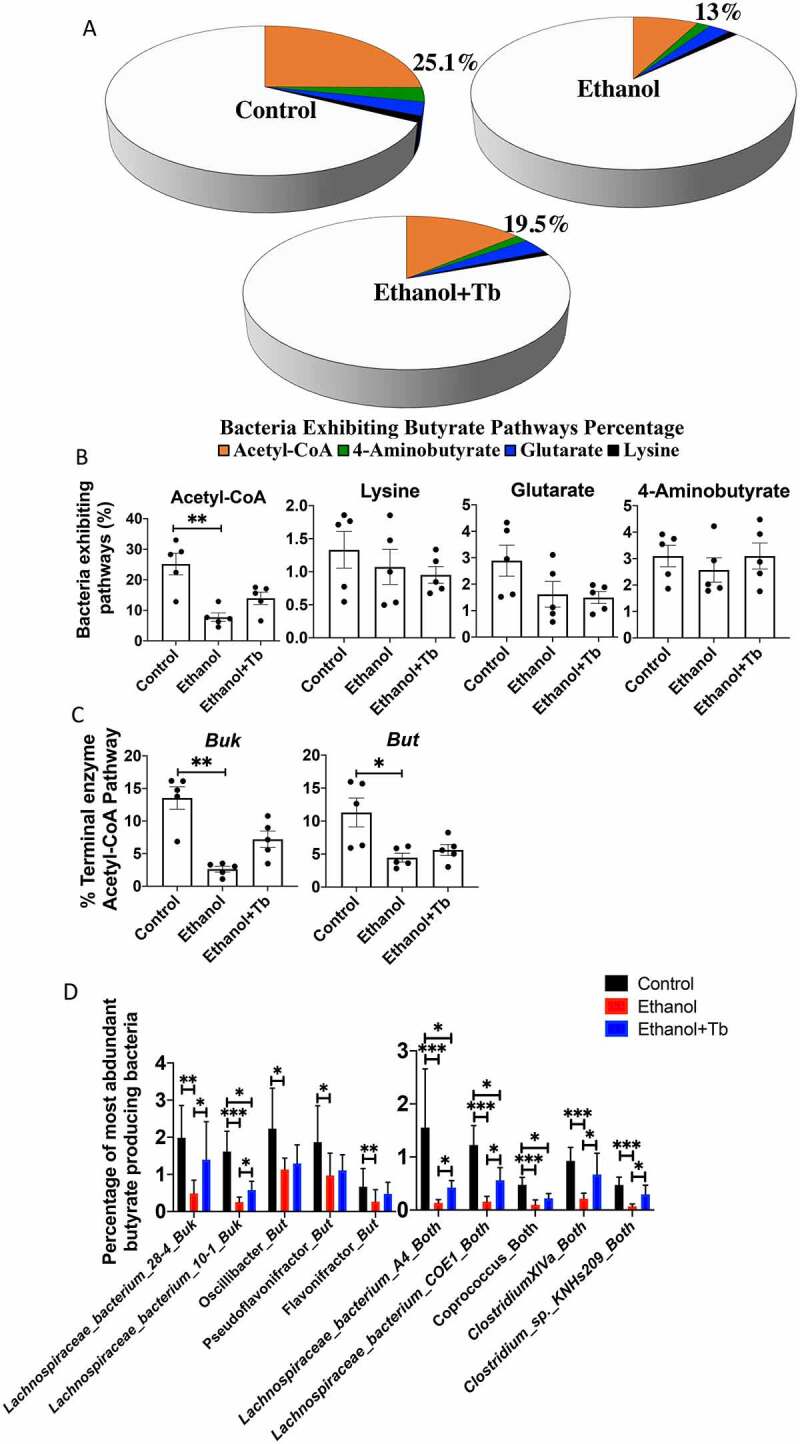
Ethanol-induced significant reduction in butyrate synthesizing pathways. WGS analysis was conducted for all three treatment groups (Control, Ethanol and Ethanol +Tb). A& B) Percent abundances of the bacteria exhibiting butyrate pathways in the microbiome and C) Percent abundances of two major terminal enzymes *but-* butyryl-CoA: acetate-CoA transferase and *buk –* butyrate kinase observed in acetyl-CoA pathway and D) Percentage of most abundant butyrate-producing taxa (closest references) expressing – *buk, but* or both genes. All data represented as mean ± SEM for n = 5 mice in each treatment groups along Kruskal Wallis corrected *p*-value, *p* < .05 = *, *p* < .01 = **, *p* < .001 = ***

Following PICRUSt analysis that provided putative association of butyrate-synthesizing genes with associated taxa, we employed WGS sequencing to quantify and validate the effect of ethanol on butyrate-synthesizing pathways. Percent abundances of butyrate-producing bacteria were calculated as a percentage of total bacterial genomes exhibiting butyrate synthesizing pathways using the database of butyrate producers provided by Vital et al. 2017.^15^ The results demonstrate that in the Control group mice, the microbiome is comprised of 25.1% bacteria harboring the acetyl-CoA pathway for butyrate synthesis followed by 3.1%, 2.9%, and 1.3% that have the potential to produce butyrate via the 4-aminobutyrate, glutarate, and lysine pathways, respectively (Figure 4a). Bacteria exhibiting the acetyl-CoA pathway were most affected by chronic ethanol feeding and their decrease in abundance was partly prevented by Tb administration (Figure 4b). Within the acetyl-CoA pathway, the two major terminal enzymes required for converting butyryl-CoA to butyrate are encoded by *but –* (butyryl-CoA: acetate CoA transferase) and *buk* – (butyrate kinase) and can serve as biomarkers for identifying butyrate-producing bacteria were affected by ethanol (Figure 4c).^16,17^ Analyses on individual taxa further revealed that groups exhibiting but (e.g., *Oscillibacter, Pseudoflavonifractor*, and *Flavonifractor)*, buk (e.g., *Lachinospiraceae_bacterium_28.4* and *Lachinospiraceae_bacterium_10-1*), and both enzymes (*Lachinospiraceae_bacterium_A4, Lachinospiraceae_bacterium_COE1, Coprococcus, Clostridium Cluster XVIa*, and *Clostridium_sp_KNHs209*) were affected. Ethanol-induced loss was prevented with Tb administration for most of these butyrate-producing taxa (Figure 4d).

## Steatohepatitis and injury induced by ethanol-mediated loss of butyrate-producing bacteria are attenuated by tributyrin

**Figure 5.**
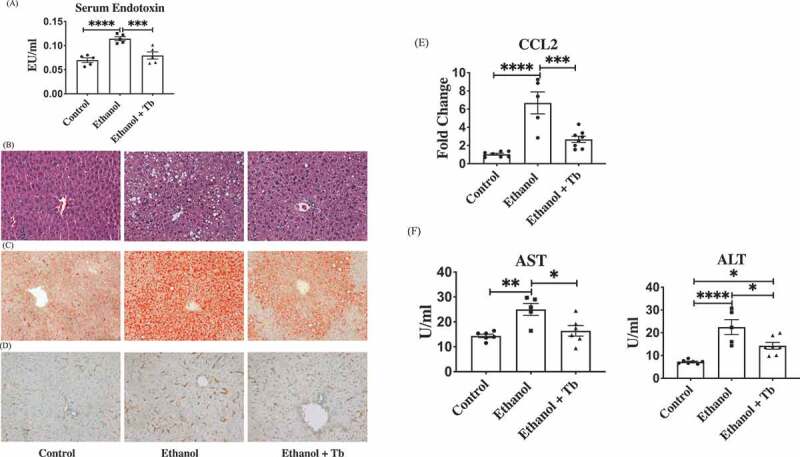
Tb attenuates ethanol-induced alterations in the gut and liver. A) Analysis of gut permeability by measuring serum endotoxin levels (EU/ml), B) Micrographs depicting evaluation of both macro and micro vesicular hepatic steatosis through H&E staining, C) Micrographs depicting hepatic lipid accumulation through oil-red-o staining, D) Micrographs depicting hepatic inflammation through F4/80^+^ staining for Kupffer cells/macrophages, E) Gene expression of pro-inflammatory chemokine CCl2 (fold change) and F) Level (U/ml) of markers of hepatic injury, ALT – Alanine transaminase and AST – Aspartate transaminase. Note: All data represented as mean ± SEM for n = 5 mice n each treatment groups along Kruskal Wallis corrected *p*-value, *p* < .05 = *, *p* < .01 = **, *p* < .001 = ***, *p* < .0001 = ****

In addition to attenuating the decrease in butyrate-producing bacteria, Tb administration significantly prevented the ethanol-induced increase in serum endotoxin levels indicative of intestinal permeability and microbial translocation (Figure 5a). Further, we also examined the effects of Tb on hepatic steatosis, inflammation, and injury, which are the major pathological consequences of alcohol-induced gut dysbiosis. The data showed that Tb, in association with the prevention of loss of butyrate-producing bacteria, significantly attenuated both macro and microvesicular steatosis (H&E staining), hepatic triglyceride levels, and lipid accumulation (oil-red-o staining) (Figure 5b-C). In association with hepatic steatosis, there was also a significant decrease in ethanol-induced hepatic inflammation in Ethanol+Tb treated animals. Specifically, Tb administration decreased F4/80+ Kupffer cells/macrophages (Figure 5d) and gene expression of pro-inflammatory chemokine CCl2, relevant for ALD (Figure 5e). Moreover, the increase in markers of hepatic injury alanine aminotransferase (AST) and aspartate aminotransferase (ALT) caused by ethanol feeding were also significantly attenuated by Tb administration (figure 5f).

## Discussion

The main goal of the present study was to elucidate the effects of chronic ethanol consumption on the functional consequences driven by microbial dysbiosis that would be relevant for ALD pathogenesis. It is increasingly recognized that for restoring the functionality of the gut-liver axis, it is essential to profile the alcohol-induced changes in the gut-microbiome along with the characterization of the related bacterial genes and metabolite composition. Work done by others and us indicates that alcohol-induced functional dysbiosis, likely involves a decline in SCFAs, particularly butyrate, which contributes to the development of hepatic steatosis, inflammation, and injury.^9,12^ Importantly, butyrate is a primary fuel source for colonocytes that maintains tight junction proteins, gut-barrier integrity, intestinal homeostasis, and proliferation of hepatocytes.^7^ Butyrate also exhibits anti-inflammatory activity in the intestinal lamina propria and regulates gene expression by inhibiting histone deacetylase.^12,18^

In the present work, the initial taxonomic assessment of the mice's intestinal butyrate-producing bacterial communities, inferred from 16S rRNA gene sequencing, revealed that chronic ethanol feeding led to a significant decrease in phylum *Firmicutes*, that encompasses the majority of butyrate-producing bacteria belonging to distinct families within the phylum. Importantly, we further leveraged the well-substantiated inventory of butyrate-producing genera^15^ together with our metagenomic analysis, to define microbial butyrate-producing communities within *Firmicutes* families, that were decreased by chronic ethanol exposure. In particular, we identified that ethanol-induced dysbiotic effects predominantly led to a decrease in the butyrate-producing bacterial genera within the *Lachnospiraceae* family, which represents the largest consortium of butyrate-producing bacteria in the phylum *Firmicutes*.^15^ In comparison, other known butyrate-producing families did not show a significant decline as an aggregate; however, several specific butyrate-producing genera comprised within them were significantly decreased by chronic ethanol feeding. Clinical studies on severe alcohol-induced cirrhosis and alcohol-associated hepatitis patients have also reported a decline in *Lachnospiraceae and Ruminococcaceae* families.^19,20^ This supports the clinical relevance of the findings in our preclinical animal model that comprehensively details the effects of chronic ethanol exposure on butyrate-producing genera.

Following 16S rRNA gene sequencing analysis, whole genome shotgun sequencing (WGS) analysis further provided a more in-depth and detailed taxa assessment and abundance estimation of the butyrate producing bacteria affected by ethanol, in the C57BL/6 N mouse gut microbiome. Interestingly, these data demonstrated inherent differences in the type of butyrate producing bacteria in humans and control C57BL/6 N mouse gut microbiome. In particular, major butyrate producing species, commonly found in the human gut microbiome, e.g.,, *Eubacterium rectale, Faecalibacterium prausnitzii, and Clostridium leptum*^15,16,21,22^ were not observed in the C57BL/6 N mouse gut microbiome and have also not been reported in earlier studies. However, importantly, these data demonstrated that the observed decrease in butyrate-specific bacteria in the C57BL/6 N mouse gut microbiome, across multiple bacterial families i) constitutes a major component of the loss of overall diversity that progressively declines over time following chronic ethanol exposure and ii) corresponds with the loss of butyrate synthesis function as indicated by the decrease in cecal butyrate levels.

Notably, 16S rRNA gene and WGS metagenomic analyses comprehensively detailed not only the butyrate-producing microbial communities but also their associated butyrate metabolic pathways, that were affected by chronic ethanol feeding. Earlier work has demonstrated that in the context of butyrate-producing microbial communities, targeting complete pathways is a more compelling way to predict the butyrogenic function.^15^ Accordingly, our metagenomic analysis of the overall butyrogenic function of the gut microbiome identified acetyl-CoA pathway as the main pathway for butyrate production that was significantly decreased by ethanol. Further, the effects of ethanol leading to the reduction in acetyl-CoA pathway is a highly significant and relevant component of the loss of butyrate production and functional dysbiosis of the microbiome because i) acetyl-CoA pathway predominates butyrate production as compared to lysine, glutarate, and 4-aminobutyrate pathway and ii) reduction in acetyl-CoA pathway cannot be sufficiently compensated by an increase in expression and abundance of other pathways.^15,16^ It is noteworthy that the mice microbiome share similarities with the human microbiome, which also comprises acetyl-CoA as the main butyrate synthesizing pathway harbored by the majority of butyrate-producing bacteria that drives the butyrate production.^16^ Taken together, these findings have provided a metabolic framework that is an essential initial step in investigating the role of the compromised butyrogenic function in ALD pathogenesis. Moreover, the WGS metagenomic analysis demonstrated that there was an ethanol-induced decrease in the abundance of *but* and *buk* genes, encoding the key terminal condensing butyrate enzymes of the acetyl-CoA pathway^16^ (Supplemental Figure S3). Employing this critical information, percent of the most abundant taxa harboring *but* and *buk* genes were also identified.

The pathogenic role of the ethanol-induced loss of butyrate-producing bacteria and butyrogenic function in ALD was strongly supported by the hepatoprotective effects of Tb supplementation. In ALD, the role of gut-liver axis is well-established for the development and progression of the disease. Our recent work has shown that oral administration of Tb increases the concentration of butyrate in the hepatoportal vein, which exerts a protective effect on the liver by modulating pathogenic epigenetic mechanisms involved in ethanol-induced hepatic steatosis and injury.^12^ Importantly, the current study revealed that the ethanol-induced decrease in certain butyrate-producing bacteria was significantly attenuated by Tb supplementation over the study period of 7 weeks. These data indicate that Tb supplementation over a longer period has the potential to further increase the population of several other butyrate producers, leading to an increase in the key butyrate synthesizing genes (*but* and *buk*) and consequently the abundance of acetyl-CoA utilizing bacteria. This influence and protective effect of Tb – a butyrate prodrug on the butyrate-producing microbial community was an unexpected finding since it was provided to alleviate the butyrate deficiency and hepatic effects of alcohol consumption. Hence, this study has revealed a novel hepatoprotective role of Tb that also involves mitigation of ethanol-induced gut dysbiosis.

In conclusion, our findings establish that the loss of butyrate-producing bacteria and butyrate genes is a hallmark feature of ethanol-induced microbial dysbiosis and plays an important causal role in the development of the alcohol-associated liver disease. Importantly, this study identified the relevance of butyrate-producing bacteria that can be used as a potential probiotic in the treatment of alcohol-induced gut dysbiosis and the development of liver disease. Additionally, further research can be directed toward the development of Tb as a major component of a nutrition-based, preventative treatment strategy for ALD. The future direction of this research will be the identification of other relevant bacterial species in conjunction with functional outcomes affected by alcohol for the development of targeted therapeutic strategies for ALD.

## Materials and methods

### Animals

All animal experiments were performed according to the criteria outlined in the Guide for Care and Use of Laboratory Animals and with the approval of the University of Louisville Animal Care and Use Committee. For this study, eight-week-old male C57BL/6 N mice were obtained from Harlan, Indianapolis. Mice were housed in a pathogen-free, 12-hour light/12-hour dark cycles and temperature-controlled animal facility accredited by the Association for Assessment and Accreditation of Laboratory Animal Care. Mice were pair-fed Lieber-DeCarli liquid diet containing either ethanol (Ethanol) or isocaloric maltose dextrin (Control) for 7 weeks. Ethanol was gradually increased for 1 week and then mice were fed the ethanol diet [5% (v⁄v)] ad libitum for the next 7 weeks. A subset of ethanol-fed mice was administered tributyrin (Sigma Aldrich, St. Louis MO) by oral gavage (2 g/kg and 5 days per week) for 7 weeks. Fecal samples were collected for metagenomic analysis, and blood and liver tissues were harvested for various biochemical analyses.

### Liver histology and immunostaining

For histological analysis, liver sections were fixed in 10% buffered formalin for 24 h and embedded in paraffin tissue sections stained with hematoxylin-eosin (H&E) and examined under light microscopy. To examine the amount of fat accumulation, the liver sections were stained with Oil-Red-O. Frozen liver sections were washed in phosphate-buffered saline twice for 5 min. Oil-Red-O and 85% propylene glycol were added with agitation for 15 min, followed by washing in tap water. Macrophage infiltration was determined by F4/80 staining protocol using the manufacturer’s instructions (Abcam, Cambridge, MA).

### Serum analysis

Blood samples were allowed to stay at room temperature for 40 min and centrifuged at 1,500 g for 10 min at 4°C. The supernatant was transferred to a new tube and centrifuged for 5 min at 10,000 g. Endotoxin levels were measured with Limulus Amebocyte Lysate kit (Lonza, Walkersville, MD) according to the manufacturer’s instructions. Serum Alanine Aminotransferase (ALT) and Aspartate Aminotransferase (AST) were measured according to the manufacturer’s instructions (Infinity™ ALT and Infinity™ AST; Thermo Scientific, Waltham, MA).

### Quantitative real-time PCR analysis

Total RNA was isolated using TRIzol Reagent (Invitrogen, Carlsbad, CA), and qRT-PCR was performed as previously described.^12^ The relative gene expression was analyzed using the ΔΔCt method by normalizing with β-actin gene expression in all the experiments and is presented as a fold change over the control treatment group, which was set at 1. The mouse CCL2 mRNA primers were as follows: forward: GGCTCAGCCAGATGCAGT; reverse: TGAGCTTGGTGACAAAAACTACAG; and mouse β-actin mRNA primers were as follows: forward: CAGCTGAGAGGGAAATCGTG, reverse: CTCCAGGGAGGAAGAGGATG.

### Measurements of butyrate levels

The cecal content was extracted and derivatized with pentafluorobenzyl bromide (PFBBr). After incubation for 15 min at 60°C reaction products were extracted with hexane and analyzed by GC-MS. The standards/samples were analyzed on Agilent DB-225 J&W GC column with a temperature gradient from 50°C to 220°C at 30°C/min at a flow rate of 1.5 ml/min with chemical ionization used as an ion source. The butyrate levels were quantified using peak area ratios, based on 8 point-standard curves.

### 16S rRNA-gene analysis

DNA was extracted from fecal samples using the QiAMP fecal DNA extraction kit (Qiagen, Maryland, MD). Metagenomic analysis of the gut microbiome was performed by analyzing the fecal DNA by amplification of the V3–V4 regions of the 16S rRNA gene using fusion primers 341 F and 785 R as previously described.^23^ Amplicon libraries were purified using SPRI beads on a Biomek liquid handler. DNA was quantized by Quant-iT PicoGreen assay, normalized, pooled, and followed by large-scale parallel sequencing on the Illumina MiSeq platform. The sequencing data consisted of an average of 141,107 merged paired-end reads per sample using a quality filtering cutoff of 30. The operational taxonomic units (OTUs) table was generated by taxonomic binning via the 1.91 version of Quantitative Insights into the Microbial Ecology (QIIME) and its various components using default settings unless otherwise noted.^24–26^ Closed reference OTU picking was performed using OTUs with 97% similarity to the clustered 13_8 release of the Greengenes reference database packaged with QIIME.^27^ To maximize the sensitivity of detecting microbial taxa, the sequence reads after quality filtering were input into the RDP classifier to generate a table of taxonomic abundances in each sample.^28^ Default settings were used with the RDP Classifier except for the confidence cutoff which was set at 0.5 and the format which was set as fixrank. An even sequence sampling depth was utilized when generating all diversity measures. Alpha diversity (within-sample species richness) was determined using multiple sub-sampling to generate rarefaction plots of unique operational taxonomic units (OTUs) per total number of sequences per sample. Further, QIIME output was generated for i) Alpha diversity measured Chao1 index (abundance of predicted OTUs/species in a sample) and Shannon index (species richness and evenness of taxa in a sample), and ii) Beta diversity weighted unifrac distance measures were utilized to test changing microbial diversity and composition among the treatment groups.^29^

### Statistical analysis

All statistical tests were performed using nonparametric tests such as the Mann–Whitney U test and the Kruskal–Wallis test with corrections (Dunn’s multiple comparisons adjusted *p*-value and Bayesian q-value correction).^30^

### PICRUSt analysis

Phylogenetic Investigation of Communities by Reconstruction of Unobserved States (PICRUSt) was used for predictive analysis of butyrate synthesis pathway.^14^ This computational approach uses 16S rRNA marker gene data in combination with a table of gene copy numbers of enzyme families present within each sequenced archaeal and bacterial taxonomic group in the IMG (Integrated Microbial Genomes) database. The output consists of the counts of coding genes from the Kyoto Encyclopedia of Genes and Genomes (KEGG). PICRUSt was also used to generate output showing the contribution of each OTU to each KEGG gene. Default settings of PICRUSt were used except during the generation of OTU contributions to genes where only butyrate synthesis coding genes were retrieved.

### Whole genome shotgun analysis

For WGS, genomic bacterial DNA (gDNA) extraction methods were optimized based on a standard protocol to maximize the yield of bacterial DNA from specimens.^31,32^ Samples were quantitated with a BR DNA qubit kit and the integrity of samples was tested on 0.8% Agarose gel. Samples were sequenced on HiSeq PE 125 using chemistries that yielded paired-end reads. Raw reads were blasted to all reference sequences from using DIAMOND.^15,33^ Only the top hits displaying a minimum length of 20 amino acids and 70% similarity to a reference recorded. Follow-up analyses on the overall pathway abundances and taxonomic composition of the acetyl-CoA pathway were done as described earlier.^15^

## Supplementary Material

Supplemental Material

## Data Availability

The data that support the findings of this study are available from the corresponding author [SB] upon reasonable request. https://louisville.app.box.com/folder.
